# Social Media Impact of Myopia Research

**DOI:** 10.3390/ijerph19127270

**Published:** 2022-06-14

**Authors:** Cristina Alvarez-Peregrina, Cesar Villa-Collar, Clara Martinez-Perez, María Ibeth Peñaloza Barbosa, Miguel Ángel Sánchez-Tena

**Affiliations:** 1 Department of Optometry and Vision, Faculty of Optics and Optometry, Universidad Complutense de Madrid, 28037 Madrid, Spain; masancheztena@ucm.es; 2 Faculty of Biomedical and Health Science, Universidad Europea de Madrid, 28670 Madrid, Spain; villacollarc@gmail.com (C.V.-C.); mariaibeth2008@gmail.com (M.I.P.B.); 3 ISEC LISBOA—Instituto Superior de Educação e Ciências, 1750-179 Lisboa, Portugal; clara.perez@iseclisboa.pt

**Keywords:** myopia, bibliometrics, altmetrics social media, research impact, infodemics

## Abstract

Background: Myopia has become a public health issue worldwide. The fast increase in myopia prevalence in the last years has been accompanied by an increase in information through social and conventional media. This has led to the fight not only against a pandemic but also against the infodemic. The excess of information has made it increasingly difficult for health professionals to identify high-quality articles. Alternative Metrics are useful tools to identify publications that provoke attention to society. This research aims to study the impact that research on myopia has had on social media. Methods: Almetric Explorer was used to make a search using “myopia” as a keyword. The 100 outputs with the highest attention were analyzed and correlated with the number of cites on Web of Science using Spearman’s correlation coefficient. Results: The top 100 Altmetric Attention Score were published in 47 journals and had a mean value of Altmetric Attention Score of 437.61 ± 718.33. The outputs were mostly discussed on Twitter, with a mean of 296.36 ± 1585.58 tweets and retweets, and a mean of 185.18 ± 211.57 readers in Mendeley. There was a low correlation between Altmetric Attention Score and Web of Science Cites for the top-100 outputs. Conclusions: although myopia is a research topic with a high interest in society, most cited articles are not those with the most impact on social media. Myopia researchers should make more effort in promoting their goals, and social media is a useful tool to share them.

## 1. Introduction

The news media allows people to get more opportunities to find information and news about society and the world. Internet news and social media expose people to all kinds of information even if they have not intentionally searched for it [1]. Malicious and abusive behaviors on social media have caused great concern because they can negatively impact personal and collective life [2]. In this way, the increase in information, especially on epidemic and pandemic diseases, has challenged researchers to attend to the detection, evaluation, and response to rumors, and their impact on public health [3].

Myopia has already become a public health issue worldwide, with estimations of 50% prevalence by 2050 and close to one billion people suffering from high myopia by then, which could decrease if preventive measures and treatments to control myopia are implemented [4,5,6]. In the last 10 years, with the increase in the prevalence of myopia, mainly in children, there has been an increase in information, exchanged through social networks, about how to try to prevent it. This is due to the desire of parents to prevent children from presenting with high myopia in the future [7]. All this has generated an infodemic, since a significant amount of misleading information and advertisements have been published. At first, the diversity of information could be considered a positive, in that the population can be better informed, especially since the diversity of information in the media environment encourages knowledge of the most important public issues [8]. However, in times of crisis the importance of this factor increases. In addition, information from social media is a key factor for society. That is, most people tend to rely on social media to understand the environment in which they live and make decisions [9]. The myopia pandemic is no exception, and due to the need for information, infodemics can originate. Infodemics are defined as situations in which undisciplined, low credibility, false, misleading, and unverified information is disseminated [10].

Although research in this field is increasing, it is important to know how the results of this research are reaching the population. 

In this sense, Altmetric.com tracks the online shares and commentaries to provide critical, real-time insights about the research produced. Altmetrics.com provides a new level of visibility into the varied interactions that occur every day with patients, patient advocates, Healthcare Professionals (HCPs), research clinicians, companies, and other researchers [11].

Although the number of scientific publications related to myopia is increasing every year, the impact of these publications is measured with bibliometric indicators that provide information about the number of citations in other papers, views, or full-text downloads [12]. These publications are usually read by other researchers, so the usefulness of scientific knowledge is limited to the degree that the knowledge is not communicated to other people. However, scientists have a growing interest in communicating their findings to society [13], and social media is becoming a great way to communicate worldwide. 

In 2010, Priem defined alternative metrics or “altmetrics” to study the impact of research on social media [14]. Altmetrics are a useful tool to identify publications that provoke attention [15]. These new metrics will allow us to analyze the quality of all the information published about myopia on social media. This is relevant, since nowadays, due to the increased prevalence of myopia in children, parents are increasingly looking for information on the Internet and social media about how to try to stop it and how it can affect lifestyles. For instance, parents and teachers can help to slow the progression of myopia by increasing the time of exposure to sunlight [16]. Altmetrics allow for measuring the impacts of articles according to the number of mentions in different online sources. This makes it possible to assess the influence of a publication in society based on traditional citations. That is, it offers the dissemination of articles to the general public. Therefore, these new analyses allow society to know the quality of information that appears on social media and thus help people to recognize true and quality information. In other words, they help avoid the increase of the infodemic.

Altmetric.com is a subscription-based tool that shows the impact of research to its authors and readers in a very graphic and new way. It allows one to monitor, search, and measure all the conversations about the articles of a magazine, as well as those published by its competitors, and collect mentions of scholarly articles from all over the web by collecting mentions from newspapers, blogs, social media, and other websites. In a matter of minutes, it allows the author to have the altmetric data to display on their platform or application. The Altmetric.com algorithm calculates an overall score based on volume, source, and author based on the mentions a document receives. This includes mentions of scholarly articles on social media sites, for example: Twitter, Facebook, Pinterest, Google+, science blogs, many mainstream media outlets, including The New York Times, The Guardian, non-English language publications such as Die Zeit and Le Monde, special interest publications like Scientific American and New Scientist, and reference peer review sites like Publons.

The algorithm collects mentions of scholarly articles from all over the web by collecting mentions from newspapers, blogs, social media, and other websites. In a matter of minutes, it allows the author to have the Altmetrics data to insert and display in their platform or application. Today the Altmetric database contains citations from over four million research sources (including journal articles, databases, images, documents, reports, and more), and is constantly growing.

This research aims to study the impact and identify the most influential myopia research articles.

## 2. Materials and Methods

### 2.1. Database

The bibliography search was carried out on 24 January 2022, through the Web of Science (WOS) database, using “myopia” as the keyword and without limitations on the rest of the parameters. This data was then analyzed using the Almetric Explorer system (Altmetric.com), which tracks the attention that research outputs such as scholarly articles and datasets received online.

All the publications obtained from the search were included in the study. The 100 with the highest attention according to Altmetric Attention Score (AAS) were analyzed by two researchers that excluded irrelevant results (Publications without AAS score or citations). The most frequently mentioned articles were selected using the AAS provided by Altmetric.com. Almetric provided the most comprehensive data and covers the vast majority of social media activity associated with scientific papers. The AAS reflects a weighted total of the mentions of the article by the different online platforms (Table 1). 

That is, if a Facebook post is selected for an article, the score would increase by 1. However, if three more Facebook posts are selected for that same article, the score is still 1. It should be noted that a simple sum of mentions is not enough to calculate the AAS. Other factors are taken into account, such as duplicate tweets or level calculations for different types of news sources [17]. Thus, the higher the AAS score, greater the impact and diffusion in society.

In the analysis, the number of articles with the highest AAS was selected as the average of the most cited and most downloaded articles of each journal (after rounding the average number of each journal). Articles were classified based on the field of research in the methods section and the number of readers in Mendeley was analyzed.

The analysis was carried out with the 100 articles with the highest AAS; thus, the AAS was greater than 5 as suggested by Kolahi [18] to achieve a more rigorous sample size. That is, with a single self-tweet it is possible to obtain an AAS = 1, and carrying out a detailed analysis of these articles could cause significant distortion of the results. 

Articles with higher AAS were then identified by an Altmetric Explorer search (Altmetric LLP, London, UK).

### 2.2. Data Analysis

Web of Science (WOS) was used for citation counts (accessed 24 January 2022) which were compared to the AAS output.

The correlation between the number of citations in WOS and data of publication with AAS was tested using Spearman’s correlation coefficient with the SPSS 25.0 software (SPSS Inc., Chicago, IL, USA).

## 3. Results

Altmetric Explorer search gave 7995 research outputs from the 23,130 indexed in the Web of Science (WOS); 6291 of these outputs have been mentioned at least once, with a total of 65,012 mentions. The first output was from 1987, with six mentions in patent databases, and the last in 2022; 2021 was the year with the highest number of outputs (*n* = 845) followed by 2015 (*n* = 576) and 2020 (*n* = 699). There was a low correlation between the date of publication and AAS (r = 0.150; *p* = 0.027). Table 2 shows the mean and standard deviation of mentions and the total number of mentions in any of the sources studied by year during the last 10 years.

Focusing on Twitter as the source with the most mentions, the three most active countries sharing information on this social network were the USA (6545 posts and 3687 profiles), Japan (3774 posts and 3499 profiles), and the UK (2835 posts and 1615 profiles). 24,417 posts and 17,888 profiles were not related to any country. Table 3 shows the most mentioned journals on different social platforms.

**Figure 1:**
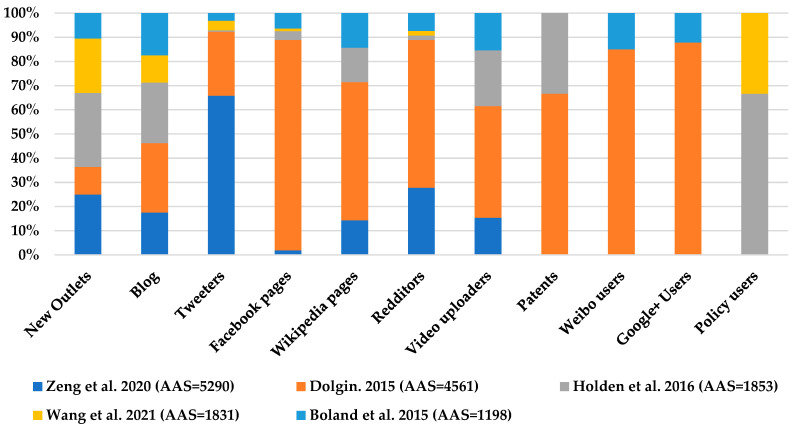
Overview of attention for the output of the five articles with the highest AAS (Own elaboration) [5,19,20,21,22].

Research outputs of the top 100 AAS were published in 47 journals and had a mean AAS value of 437.61 ± 718.33 (range from 121 to 5289). The outputs were mainly discussed on Twitter with a mean of 296.36 ± 1585.58 tweets and retweets (range from 0–14,590), and had a mean of 185.18 ± 211.57 readers in Mendeley (range from 0 to 1432). Figure 1 shows the mentions on social networks of the publications with the highest AAS values, as well as the number of readers in Mendeley.

Regarding the journals, “Ophthalmology” was the journal with the highest number of top-100 papers, with 16 papers among the top 100 Articles. This was followed by “JAMA Ophthalmology” with 10 papers, “Investigative Ophthalmology & Visual Science” with 8 papers, and “British Journal of Ophthalmology” with 6 papers.

Table 4 and Table 5 show the characteristics of the ten journals with the highest AAS attention regarding myopia research. Thus, during the years included in the study, 2420 out of 2923 items published had an AAS higher than 1 and, of these, 2344 had an AAS higher OKthan 5.

Ophthalmology had the highest cumulative AAS. The journals with the highest attraction were Ophthalmology and the British Journal of Ophthalmology with a mean AAS per published item of 27.7 and 11.5, respectively. Investigative Ophthalmology & Visual Science, Scientific Reports, and the Journal of Refractive Surgery drew online attention to 100.0% of its published articles.

Regarding the Field of Research (FoR) of the top-100 outputs, 90 were classified in division 11, “Medical and Health Sciences”, and 49 of them in group 1117, “Public Health and Health Services”. Table 5 shows the details of the main FoR.

In the analysis of the outputs that have gotten more attention, Table 6 presents the five outputs with the highest Altmetric Attention Score, as well as other traditional bibliometric parameters.

Studying the correlation between AAS and WOS cites, there was a low correlation between both values for the top-100 outputs (r = 0.221; *p* = 0.028), and there was a low correlation between AAS and WOS Cites considering the total of research outputs instead of the top 100 (r = 0.235; *p* ≤ 0.001).

## 4. Discussion

This study has analyzed how the information from research on myopia has impacted society through its dissemination on social media. Twitter stands out as the first social network with a greater number of mentions and the journal Ophthalmology has a greater AAS. However, the low correlation found between social influence and literature citation rate stands out.

In recent years, the growing interest of parents and teachers has risen in the publications of information about myopia and how to stop its progression. So, it is important to know whether society is aware of the scientific research. Compared to other areas, myopia seems to be a topic that gets a lot of attention, with a mean AAS value of 414.95 in the top-100 myopia research outputs. However, it is important to highlight that only 53 of the top-100 research outputs have been published in journals of optometry or ophthalmology. The two first research outputs according to the AAS have been published in JAMA Ophthalmology [19] and Nature [20], with 14,866 and 7276 mentions, and 28 and 323 cites in WOS, respectively. They are followed, in the third place, by the publication of Holden et al. in 2016 in Ophthalmology [5] with 513 mentions and 1094 cites in Web of Science. That could mean that journals with a wide range of areas of research would have a higher impact on society or that researchers in a specific field cite and publish in journals related to their area of research. That fact is supported by the low correlation found between AAS and the number of citations in WOS of the research outputs.

The advent of Altmetrics has allowed accurate metrics for scientific articles to spread more quickly. However, citations are still taking longer to accumulate.

It should be considered that there may be multiple confounding factors, which may influence the AAS. These can be the use of social networks by health professionals, sensationalism, or the type of publication. This leads to the AAS increasing more rapidly with respect to citations. Therefore, since citations and AAS have a different impact, in future research it would be necessary to analyze the correlation of each platform and the number of citations individually. However, it would also be necessary for all journals to include alternative metrics for their published articles in addition to traditional citations.

This would allow a greater diffusion of the research to the population. Mainly, in this field of myopia, it would allow society to know in more detail the current methods that exist to control myopia and how it increases the prevalence rate. In addition, it would also help you to know the visual hygiene measures and perform more frequently.

Altmetrics complements the traditional metric system, but it does not replace it. Researchers and publishers can see how their research is distributed on social media platforms, such as Twitter and Facebook, or through news articles. For this reason, in our study a weak correlation was found between the number of citations and AAS, it may be due to the fact that newer articles receive more attention online after publication, while the citation count may take longer to accrue. The weak correlation between the number of article citations and AAS coincides with other studies on dermatology, cardiology, or pediatric surgery [23,24,25]. This suggests that articles that have a high AAS do not have to have the same interest from researchers.

It should also be considered that there are publications of journalistic interest but not in academic research. Altmetrics reflects the attention of the online crowd, but does not reflect the quality, validity, and originality of the research. The opposite happens to traditional metrics, which focus on validity and quality, but not on the diffusion of new publications.

Analyzing the five most relevant papers in terms of AAS, the article “Association of Daily Wear of Eyeglasses with Susceptibility to Coronavirus Disease 2019 Infection” published by JAMA Ophthalmology shows that myopia was a topic with great attention due to the high AAS. To put it in context, we can compare it with the top 100 articles of Altmetric in 2019. The first one was about meta-learning of adversarial generative models with an AAS of 13,557 [26], while the first article on the subject of medical and health science was related to vaccines and autism and got the third position with an AAS of 9199 [27]. Regarding metrics, Twitter has more mentions (14,577), although News (223) also stands out. The high number of mentions may be because it was published in Medscape, which offers high visibility. The hashtag “Covid19” has been used in both social networks, which drew the attention of readers and helped spread it. This study allows readers to know that users of glasses for >8 h/day may be less susceptible to COVID-19.

In the second position is the article “The myopia boom” by Dolgin [20]. This article has allowed readers to know the rate of increase in prevalence worldwide, mainly in Asian countries, as well as risk factors and methods of treatment and prevention. Special attention has been paid to the importance of exposure to sunlight. In this way, this has been able to help parents and schools to carry out greater prevention in children.

In positions 3 and 4 are the studies that analyze the increase in the prevalence of myopia in recent years. Thus, Holden et al. [5] analyzed the increase in the prevalence of myopia worldwide. The authors obtained that in 2000, the incidence rate was 22.9%, which is estimated to have increased to 49.8% in 2050. Subsequently, Wang et al. [21], investigated how confinement has affected refractive errors in school age. Thus, the authors discovered that children between 6 and 8 years old present a significant myopic change. However, caution must be exercised in the interpretation of these results, since the study has certain limitations (use of non-cycloplegic refractions and the lack of a history of orthokeratology or ocular biometry data). Furthermore, the refractive status of younger children may be more sensitive to environmental changes than at older ages, since younger children are in a critical period for the development of myopia. These studies generate concern in society and lead to an increase in the search for information on myopia control methods. It should be noted that in these articles, the largest number of mentions come from digital newspapers in the medical area, which allows greater dissemination of the news.

Finally, in position 5, there is the article by Boland et al. [22]. In this article, the authors analyze the impact of the month of birth on the risk of developing diseases throughout life, concluding that there is an association, that is, of presenting a greater risk according to the month of birth, in 55 diseases, among which is myopia. In this case, it was the author herself who spread the news through Twitter, with the hashtags “jamiedimon” and “heart”. Scientific dissemination by authors and journals is very recent; however, this helps the population to be able to distinguish articles truthfully and reduce the risk of an infodemic.

It should be noted that in the first and last article with a higher AAS score, myopia was the secondary objective. However, it has been decided to keep these articles since the first analyzes the prevalence of myopia in Hubai, China, and the second article analyzed how the month of birth can affect the lifestyle, which is considered a risk factor for myopia. Currently, and according to the bibliometric study by Shan et al. [28], these are the topics of greatest interest to researchers. For this reason, and to include all the articles in which myopia is mentioned, the search terms have not been restricted, and only the keyword “myopia” has been used. Thus, although the article was not exclusive to myopia, it can be seen that they also have a great impact and that they can help to obtain more information in this field of research.

The years 2020 and 2021 had more publications tracking attraction, with 19,158 and 7671 mentions, respectively; 2020 is the year with the most mentions, since the article published by Zeng et al. [19], presents 14,590 mentions on Twitter. However, in terms of the number of scientific publications, 2021 was the most relevant year. These years have increased the interest of researchers to know how confinement affected refractive errors—mainly the increase in myopia. This leads to increased interest in social networks. For this reason, in this period, three articles with the most mentions analyzed the relationship between myopia or the use of glasses with COVID [19,21,29]. On the other hand, in 2021, interest in spectacle lenses for myopia control has increased. Thus, the publication by Lam et al. [30], has had a total of 239 mentions and 35 readers in Mendeley. The manufacturers of the lenses have spread this publication on Twitter with the hashtag #MiYOSMART. However, it had a greater diffusion in news. This may be because it is still a very recent topic and for now unknown by a large part of the profession. In other words, most of the dissemination has been carried out among vision professionals. However, it is very likely that in the year 2022, this publication will generate a very strong interest.

Our results agree with the study by Shan et al. [28] in which, through a bibliometric analysis, they found that the years 2021 and 2021 are the ones with the highest number of publications. This is consistent with the fact that at the same time they are the countries with the most mentions. As previously mentioned, the number of publications in the field of myopia is expected to increase in the coming years. This is due to advances in myopia control methods that are increasingly of interest to researchers. At the same time, the number of mentions will also increase, since trying to curb myopia in children is one of the issues that most worries parents today.

The years 2015 and 2016 also stand out, with 11,728 and 43,959 mentions, respectively. This is due to the attraction caused by the articles published in popular science journals such as the one published in Nature in 2015 [20] and the one published in Scientific American in 2016 [31]. This may be related to the creation of the International Institute of Myopia. This institute was created by experts from around the world, to increase research, patient management, and education in myopia. This initiative aims to prevent future vision problems and blindness associated with increased cases of myopia by organizing meetings between scientists, doctors, legislators, governments, and educators in the field of myopia to stimulate collaboration and the exchange of knowledge [32]. 

Regarding sources, Twitter is the most used, with the United States, United Kingdom, and Spain leading the profiles and tweets about myopia research. That data does not match with the countries of the institutions that published more papers about myopia which are Singapore, China, Australia, and the USA. This is due to the different reach of Twitter in these countries, with 59.3 million profiles in the USA, 16.7 million profiles in the UK, and 7.5 million in Spain vs. 6.2 million in Australia [33], or the 1.3 million users in Singapore [34]. It should be noted that despite the growing interest in trying to slow the progression of myopia in children, it is still unknown how these methods will help reduce its prevalence in the future. Therefore, the growing interest in social networks in this field of research will help to develop and improve myopia control methods, both optical and pharmacological, to reduce its prevalence and associated pathologies. That is, social networks allow us to know the most used and most adapted methods in each country and thus help vision professionals to choose the most appropriate methods in each case.

The United States is the leading country in myopia research and is one of the most mentioned in social networks, since it attaches great importance to exchanges and cooperation in the academic community, which also explains the reason for having to produce more research of high quality and with good communication and collaboration with other authors. In addition, the most cited authors belong to universities in the United States. Thus, Curtin BJ was the most cited author. The most cited article was one in which he found that high myopia was associated with abnormal proteoglycans in the sclera that changed the size and organization of collagen fibrils [35]. In turn, the most influential journals also come from the United States.

Compared to other recent studies of altimetric in health science, myopia provokes more interest in social media than in Pediatric Surgery, with a median AAS of 8 (range 0–4261) [24].

## 5. Conclusions

There is a low correlation between social influence and the citation rate of the literature, so articles should be published in journals that cover a wide range of areas of expertise used to have a greater impact on society.

This study offers information on various measures to analyze the impact of articles on social networks. It also provides important information on the dissemination of scientific knowledge of myopia control in social networks. Thus, a mean Altmetric Attention Score value of 437.61 ± 718.33 was obtained, while a mean of 296.36 ± 1585.58 tweets and retweets were obtained, and a mean of 185.18 ± 211.57 readers in Mendeley. Therefore, researchers must increase their efforts to communicate their results on social networks.

Myopia is a research topic of high interest in society, based on the results of the parameter of attention of the research outputs.

## Figures and Tables

**Table 1:** AAS depending on the online platform.

Online Platforms	AAS
News	8
Blog	5
Policy document (per source), Patent, and Wikipedia	3
Peer review (Publons, Pubpeer), Weibo, Google+, F1000, and Syllabi (Open Syllabus)	1
LinkedIn	0.5
Twitter (tweets and retweets), Facebook (only a curated list of public pages), Reddit, Pinterest, Q&A (Stack Exchange), and Youtube	0.25
Number of Mendeley readers and Number of Dimensions and Web of Science citations	0

**Table 2:** Mean and standard deviation of mentions by source and the total number of mentions that Altmetric Explorer has tracked for myopia according to year.

	Mean ± SD	2012	2013	2014	2015	2016	2017	2018	2019	2020	2021
News	0.96 ± 8.50	145	167	106	551	783	209	358	155	623	870
Blog	0.09 ± 0.74	23	26	27	84	33	15	32	13	85	52
Policy	0.05 ± 0.44	20	26	26	36	31	5	8	7	4	5
Patent	0.07 ± 0.57	54	35	32	12	11	8	0	0	4	4
Twitter	8.53 ± 213.40	450	792	927	9811	2943	1736	2410	2821	18,266	6615
Peer review	0.01 ± 0.12	3	3	1	1	1	1	0	4	9	3
Weibo *	0.01 ± 0.50	0	2	7	44	0	0	0	0	0	0
Facebook **	0.44 ± 7.20	161	151	130	878	501	180	143	79	109	96
Wikipedia	0.05 ± 0.38	23	35	23	32	13	18	9	5	14	11
Google+ ***	0.06 ± 1.96	12	9	14	195	46	10	15	0	0	0
LinkedIn †	0.00 ± 0.00	0	0	0	0	0	0	0	0	0	0
Reddit	0.02 ± 0.56	10	3	3	53	12	12	6	3	24	4
Pinterest ††	0.00 ± 0.01	0	0	0	0	0	0	0	0	0	0
F1000	0.01 ± 0.12	17	25	15	5	6	1	5	8	2	4
Q&A	0.00 ± 0.03	0	0	0	3	0	0	0	0	0	0
Video	0.02 ± 0.25	4	9	9	23	15	6	2	1	18	7
Total	10.34 ± 222.40	922	1283	1320	11,728	4395	2201	2988	3096	19,158	7671

* not trackable since 2015 ** only a curated list of public Pages *** not trackable since 2019. † not trackable since 2014. †† not trackable since 2013.

**Table 3:** Mention of the journals on the different social media.

Journal Title	News	Blog	Twitter	Facebook	Wikipedia	Google+	Video
Investigative Ophthalmology & Visual Science	351	33	1255	132	53	3	13
Journal of Cataract & Refractive Surgery	45	3	305	66	43	4	0
Ophthalmology	1161	58	1495	149	42	27	28
British Journal of Ophthalmology	301	34	1038	80	27	2	6
Optometry and Vision Science	201	11	566	114	29	9	1
American Journal of Ophthalmology	51	5	681	82	15	1	8
PLOS ONE	179	11	521	72	4	4	8
Ophthalmic & Physiological Optics	122	11	538	38	21	0	3
Journal of Refractive Surgery	18	11	461	35	33	1	2
Scientific Reports	34	5	750	18	5	2	0
Total	2463	182	7610	786	272	53	69

**Table 4:** Journals in the top-10 Altmetric Attention Score in myopia research (a) and AAS rank for each of the journals studied (b).

**(a)**						
**Journal Title**	***n***	**Number of** **Mentioned Outputs**	**Total** **Mentions**	**AAS**	**IF**	**Citations, WOS**
Ophthalmology	342	269	3431	9474	12.08	47,497
Investigative Ophthalmology & Visual Science	611	422	2097	4165	4.80	50,693
British Journal of Ophthalmology	289	248	1643	3332	4.64	15,138
Optometry and Vision Science	349	247	1057	2231	1.97	17,435
PLOS ONE	218	194	834	1730	3.24	4968
Ophthalmic & Physiological Optics	228	189	878	1459	3.12	9272
Journal of Cataract & Refractive Surgery	407	306	1090	1246	1.55	37,550
American Journal of Ophthalmology	287	229	1191	1145	5.26	25,147
Scientific Reports	159	157	823	786	4.38	1590
Journal of Refractive Surgery	233	184	622	667	3.57	24,405
Total	2923			26,235		233,695
**(b)**						
**Journal Title**	**AAS/Article**	***n*** **/AAS**	***n*** **/AAS Range**			
			**1**	**2–5**	**6–10**	**>10**
Ophthalmology	27.7	256 (69.0%)	1	14	5	236
Investigative Ophthalmology & Visual Science	6.8	422 (100.0%)	2	14	6	400
British Journal of Ophthalmology	11.5	243 (98.0%)	2	5	1	235
Optometry and Vision Science	6.4	245 (99.2%)	1	14	6	224
PLOS ONE	7.9	192 (99.0%)	0	6	6	180
Ophthalmic & Physiological Optics	6.4	187 (98.9%)	1	6	3	177
Journal of Cataract & Refractive Surgery	3.1	306 (100.0%)	0	3	0	303
American Journal of Ophthalmology	4.0	228 (98.2%)	0	2	1	225
Scientific Reports	4.9	157 (100.0%)	2	3	6	146
Journal of Refractive Surgery	2.9	184 (100.0%)	0	0	2	182
Total		2420	9	67	36	2308

*n*: Number of published items; AAS: Altmetric Attention Score: IF: Impact Factor.

**Table 5:** Field of Research of journals publishing the top-100 AAS outputs about myopia.

* Division 1	** Group 1	** Group 2	** Group 3	* Division 2	** Group 4	Outputs (*n*)
01				02		1
06	0601					1
06	0604					1
06	0604			11	1113	3
06				11		1
07	0705			14	1402	1
11						4
11	1103	1117				1
11	1103	1113	1117			5
11	1109					1
11	1113					29
11	1113	1117				8
11	1114					2
11	1117					35
11	1199					1
14	1402					2
15	1503	1505				1
17	1701					1
No FoR						2

* Divisions: 01 Mathematical Sciences; 02 Physical Sciences; 06 Biological Sciences; 07 Agricultural and Veterinary Sciences; 08 Information and Computing Sciences; 11 Medical and Health Sciences; 14 Economics; 15 Commerce, Management, Tourism, and Services; 17 Psychology and Cognitive Sciences. ** Groups: 0601 Biochemistry and Cell Biology; 0604 Genetics; 0705 Forestry Sciences; 1103 Clinical Sciences; 1109 Neurosciences; 1111 Nutrition and Dietetics; 1113 Ophthalmology and Optometry; 1114 Pediatrics and Reproductive Medicine; 1117 Public Health and Health Services; 1199 Other Medical and Health Sciences; 1402 Applied Economics; 1503 Business and Management; 1505 Marketing; 1701 Psychology.

**Table 6:** Top-5 research outputs about myopia according to the Altimetric Attention Score (AAS).

AAS	Title	Journal/Collection Title	Publication Date (dd/mm/yyyy)	Mentions	Cites in WOS
5289	Association of Daily Wear of Eyeglasses with Susceptibility to Coronavirus Disease 2019 Infection	JAMA Ophthalmology	1 November 2020	14,866	28
4560	The myopia boom	Nature	18 March 2015	7276	323
1853	Global Prevalence of Myopia and High Myopia and Temporal Trends from 2000 through 2050	Ophthalmology	1 May 2016	531	1094
1831	Progression of Myopia in School-Aged Children After COVID-19 Home Confinement	JAMA Ophthalmology	1 March 2021	1147	46
1198	Birth month affects lifetime disease risk: a phenome-wide method	Journal of the American Medical Informatics Association	2 June 2015	895	64

## Data Availability

Not applicable.
